# Effect of topical naringenin and its combination with minoxidil on enhancing hair growth in a mouse model

**DOI:** 10.25122/jml-2023-0094

**Published:** 2023-11

**Authors:** Nooralhuda Khayoon, Sarmad Gany, Najah Rayish Hadi, Ahmed AL Mudhafar

**Affiliations:** 1 Department of Pharmacology & Therapeutics, Faculty of Medicine, University of Kufa, Najaf, Iraq

**Keywords:** naringenin (NAR), minoxidil (MXD), hair loss, TAO-C, VEGF, KGF

## Abstract

This study aimed to investigate the efficacy of naringenin (NAR) in reducing hair loss. Twenty-four adult Wistar Albino mice, weighing between 25-35 g and aged 6-7 weeks, were used in this research. The dorsal hair of these mice was meticulously clipped and stained subsequently. The mice were randomly divided into four groups (n=6 for each group): (1) negative control group, treated with absolute ethanol alcohol as the vehicle (2) minoxidil (5%) treated group; (3) 0.5% naringenin treated group, and (4) naringenin plus minoxidil treated group. The treatment groups had significantly higher total antioxidant capacity in tissue levels and increased serum levels of vascular endothelial growth factor compared to the control group. No significant differences were observed in keratinocyte growth factor tissue levels between the treatment and control groups. However, the medication significantly increased hair growth, hair follicle diameter expansion, and hair follicle quantity compared to the control group. The finding suggests that the antioxidant and anti-inflammatory properties of NAR significantly reduced hair loss in adult male mice.

## INTRODUCTION

Hair loss, or baldness, is a common dermatological issue, affecting approximately 48.9% of men and 36.1% of women. Hair thinning, follicle shrinkage, and hair loss can happen in many places on the body, not just the head, and may be triggered by a psychological condition known as unfavorable squeal [1]. The process of hair loss involves two primary aspects. Firstly, disruption of the anagen phase, caused by an atypical hair cycle, leads to a rapid transition from anagen to telogen through catagen. This results in the hair shaft being shed prematurely, with hairs not remaining in the anagen phase long enough to achieve the desired length, thereby increasing the proportion of hairs in the telogen phase [2]. Secondly, a reduction in the size and function of the dermal papilla and/or hair matrix alters the diameter and characteristics of the hair [3].

Hormonal changes, medical conditions, genetic factors, drug use, anxiety, and stress are significant reasons for hair loss [4]. The most common types of hair loss are alopecia, telogen effluvium (TE), male pattern hair loss (MPHL), and female pattern hair loss (FPHL). Alopecia can be brought on by a shift in hormone levels or by an emotionally stressful situation. Most cases of MPHL can be traced back to either hormonal or hereditary factors. Androgenic factors, on the other hand, are the most common cause of FPHL. Telogen effluvium, which may be acute or persistent, is suspected when hair thinning occurs despite normal hair density. Reduced hair density may affect the entire scalp (diffuse alopecia), be localized to one or more specific regions of the head (patterned alopecia), or manifest as discrete bald spots (patchy alopecia). In cases of spotty alopecia, the scalp may exhibit completely bald areas (alopecia Areata, cicatricle alopecia) or have tiny, broken hairs (trichotillomania, hair shaft disorders) [5]. Although telogen effluvium can be caused by a number of distinct mechanisms, it is always characterized by the simultaneous transition into exogen of a large number of follicles [6]. When exacerbating factors are mitigated, or specific drug administrations are ceased, normal hair growth and regeneration typically resume [7]. TE treatments are usually divided into non-medical and medical treatments depending on the state of the disease. In acute TE, non-medical treatments primarily focus on addressing the underlying cause. This approach often does not require direct therapeutic intervention, as acute TE is usually self-limiting with a defined duration. Hair growth typically resumes naturally once the underlying cause is resolved, usually within a three to six-month period. The most important role in managing acute cases is patient education and reassurance about hair loss in telogen effluvium. Overall, hair regrowth in acute TE cases returns to full density and normal appearance within a year [8, 9]. Minoxidil, a potassium channel opener, induces vasodilation of arteries, thereby enhancing blood flow and nutrient delivery to hair follicles.

Consequently, minoxidil stimulates plasma circulation to the hair, promoting the anagen phase and reducing hair shedding during the telogen (resting) phase [10]. Oxidative stress plays an important role in hair loss, and it is defined as a state in which the body's antioxidant defense capability is inadequate in comparison to the production of oxidants, including free radicals, reactive oxygen species (ROS), and reactive nitrogen species (RNS) [11]. Damage to cell structures is a hallmark of oxidative stress, which arises when the equilibrium between pro-oxidant and anti-oxidant processes in cells is disrupted.

Reactive oxygen and nitrogen species, produced during normal cellular respiration, can have either a protective or deleterious effect on the organism [12]. The overproduction of ROS can lead to the peroxidation of various cellular components, including nucleic acids, proteins, lipids, sulfur-containing enzymes, and carbohydrates, compromising cell functions. These changes may be caused by interior processes, such as inflammation, illnesses, metabolism, or external (radiation, diet, pollution, drugs) [13]. The use of antioxidants, such as superoxide dismutase (SOD), catalase, glutathione peroxidase, vitamins E and C, and glutathione (GSH), can prevent or minimize ROS-induced damage. These agents are thought to play a crucial role in the body's restorative pathways, opposing the stress-induced release of free radicals [14]. Total antioxidant capacity (T-AOC) is a biological marker used to estimate the quantity of free radicals neutralized in biological systems through an assay solution. The efficacy of this test in predicting T-AOC is based on the reduction of copper ions from copper (II) to copper (I) [13]. Antioxidants, categorized into enzymatic and non-enzymatic agents, play a crucial role in maintaining a balance between oxidizing and non-oxidizing agents. This balance is essential to prevent the excessive accumulation of ROS and to inhibit cellular damage caused by oxidative stress [15]. Hair damage often results from increased oxidative stress due to an imbalance between free radical production and the body's ability to neutralize them [16, 17].

Angiogenesis, the growth of new blood vessels from pre-existing ones, is regulated by signaling molecules like vascular endothelial growth factor (VEGF). Although arterial endothelial cells are the primary target of VEGF, they also appear to affect other cell types, such as stimulating monocyte/macrophage migration [18]. Vascular endothelial growth factor is also known as vascular permeability factor. VEGF, epithelial growth factor (EGF), insulin-like growth factor (IGF), fibroblast growth factor (FGF), and platelet-derived growth factor (PDGF) are just some of the growth factors involved in regulating the hair development cycle. Hair loss occurs when these growth hormones are improperly regulated [19-21].

Keratinocyte growth factor (KGF), also known as fibroblast growth factor 7 (FGF7), is produced by fibroblasts in the skin and acts as a paracrine growth factor for epidermal keratinocytes. The same tyrosine kinase receptor is shared by keratinocyte growth factor and acidic fibroblast growth factor. Tumor growth factor-alpha, epidermal growth factor, acidic and basic fibroblast growth factor are all well-established epidermal mitogens, but keratinocyte growth factor's stimulatory effects on DNA synthesis in cultured keratinocytes are two to ten times greater [22]. It has also been reported that KGF mRNA levels can increase approximately 160-fold from their baseline levels following skin injury [23]. KGF, known for promoting epithelial migration and proliferation during the healing process, is likely produced in response to the local release of interleukin-1 (IL-1) and serum factors. Furthermore, KGF has been identified as essential for hair cycle growth, particularly in stimulating hair follicle morphogenesis [24, 25].

The compound naringenin (NAR) is a member of the flavanones family and is found in many foods. Its glycoside form is abundant in foods like citrus products, bergamot, and tomatoes. NAR, chemically known as 4′, 5, 7-trihydroxyflavonone (C15H12O5), is synthesized from the aromatic amino acid phenylalanine [26]. It exists in several forms, including aglycone, neohesperidoside, and glycosylated forms. Naringin, the inactive glycone form of naringenin, is consumed and quickly hydrolyzed by the gut bacterial naringinase enzyme to yield rhamnose and naringenin (active aglycone form). It has been shown that naringenin is quickly bioavailable in circulation after being absorbed by the intestines. Although naringenin is poorly soluble in water, it is soluble in organic solvents like alcohol, which affects its total bioavailability [27]. The pharmacological impact of naringenin is extensive, including anti-cancer properties, insulin-like actions in diabetes treatment, and antioxidant and anti-inflammatory effects in hypertensive conditions. Additionally, it exhibits anti-mutagenic, anti-proliferative, anti-fibrogenic, anti-atherogenic, antimicrobial, anti-atherosclerotic, neuroprotective, antidiabetic, immunomodulatory, hepatoprotective, and cardioprotective properties. These pharmacological effects of naringenin have been linked to the compound's ability to scavenge free radicals, which are produced in response to different metabolic circumstances [28]. This study aimed to investigate the efficacy of naringenin in reducing hair loss.

## MATERIAL AND METHODS

### Study location and animals

**Figure 1:**
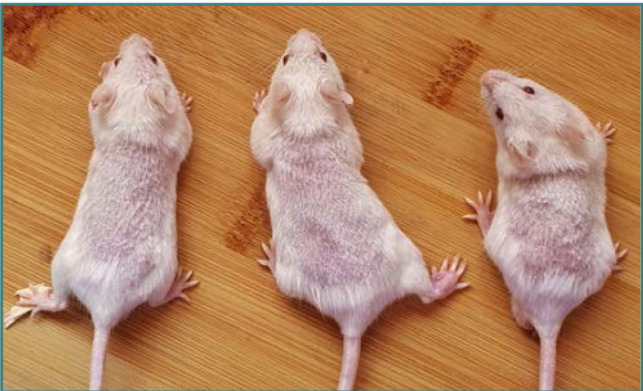
Clipped dorsal coat of Wistar Albino mice

The research was conducted at the Faculty of Medicine, University of Kufa, within the pharmacology and therapeutics section and the Middle Euphrates Unit for Cancer Research. The Animal House at the College of Science provided 24 adult Wistar Albino mice, aged between 7-8 weeks and weighing approximately 25-30 grams. These animals were accommodated in a controlled environment, with a stable temperature of 22°C±2°C) and unrestricted access to water and food. After two weeks of acclimatization, the dorsal hair of the mice was carefully removed using an electric shaver a day before the experiment, and diethyl ether was used as an anesthetizing agent. The hair removal was performed gently to ensure the targeted area was cleared without causing harm to the skin, which appeared pinkish post-shaving, indicating the resting phase of hair growth in the treated area [29] (Figure 1).

**Figure 2:**
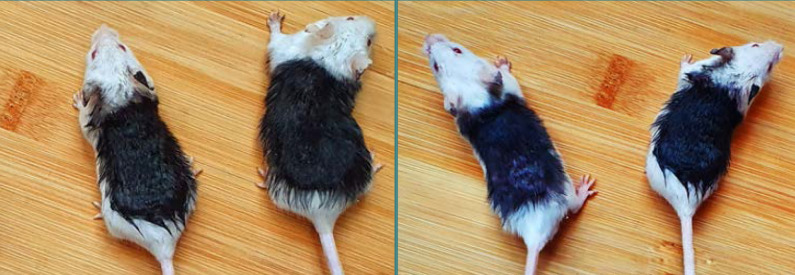
Post-staining visualization of mice dorsal skin

After the clipping procedure, the exposed dorsal skin of the mice appeared rosy in color. To facilitate differentiation between the newly growing hair and the hairless areas, Hoffmann's commercial dye was applied to the hairless dorsal skin of the mice. Then, ethanol was used to rinse the staining region. The purpose of this staining process was to distinguish between the areas of new hair growth and the regions that remained hairless. This distinction was essential to quantify the proportion of newly grown hair (white region) and the hairless (black region) areas [30, 31] (Figure 2).

Then, the mice were randomized into four groups (n=6) as follows:

1. Ethanol (negative control) group (n=6): Mice received 0.4 ml/kg/day of absolute ethanol alcohol applied topically for 21 days. The ethanol was administered by a micropipette to the hairless skin. Subsequently, skin tissue samples were collected for analysis.
2. NAR treated group (n=6): Mice received 0.5% NAR dissolved in absolute ethanol alcohol at a dosage of 0.4 ml/kg/day, applied topically for 21 days.
3. Minoxidil (active control) treated group (n=6): Mice received 5% minoxidil (0.4ml/kg/day), applied topically for 21 days.
4. NAR+minoxidil (MXD) treated group (n=6): Mice received an equal amount of 0.5% NAR and 5% minoxidil solution (0.4ml/kg/day via topical route) for 21 days. After that, the skin tissue for each group was collected.

### Preparation of skin tissue samples for histopathology

On the 22^nd^ day of the experiment, following the removal of dorsal hair, a 5 mm skin sample was excised from each mouse. After flattened, the tiny skin sample was fixed in 10% formaldehyde, normal histological procedures were performed, and paraffin blocks were created. The skin tissue sections from the deparaffinized and rehydrated mice were stained with 1% eosin and hematoxylin at room temperature for histological examination. Microscopical comparisons of control and treatment groups were made by counting and measuring hair follicles in tissue sections.

### Examination of histological sections

The hair follicles in all four groups (control and treated) were meticulously examined using a light microscope. This analysis focused on two primary characteristics:

1. **Follicle count analysis:** We quantified the hair follicles using a light microscope set to a high-power field (x100). Each histological section, sized around 10 mm, was systematically divided into four quadrants using a calibrated ocular lens. The follicles in each quadrant were counted, and the average number per section was computed. This approach allowed for a comparative analysis of follicle density between the control and treated groups [32, 33].
2. **Follicle diameter measurement:** To assess the size of the hair follicles, we randomly selected five follicles from each group for detailed examination under higher magnification (x400). The diameter of each follicle was measured both longitudinally and transversely, with the results expressed in micrometers. This measurement provided insights into the average follicle size within each group, facilitating a comparison between control and treated mice [32, 33].

### Skin tissue preparation for ELISA measuring of TAO-C, VEGF, and KGF levels

On the 22^nd^ day of the experiment, mice were anesthetized using intraperitoneal injections of xylazine (10 mg/kg) and ketamine (100 mg/kg). Subsequently, a punch biopsy of the skin was performed through a midline incision for each mouse. Afterward, the skin tissue was rinsed with normal saline to remove red blood cells or clots and stored in a deep freeze at -80 C° for enzymatic analysis. For the biochemical assays, the skin tissues were first weighed and then homogenized using a high-intensity liquid processor in a solution containing 1:10 (w/v) phosphate buffer saline (pH 7.4) that contained 1% triton X-100 and a protease inhibitor cocktail. The homogenate was centrifuged at 3,000 rpm and 4°C for 20 minutes. The resulting supernatant was collected to quantitatively assess T-AOC, VEGF, and KGF levels using a commercially available ELISA kit, according to the manufacturer’s instructions.

### Statistical analysis

Graph-Pad Prism, version 9.0.0, was used for all data processing and graphical representation. (San Diego, California, USA). The Shapiro-Wilk test was applied to confirm that the data followed a normal distribution. A one-way analysis of variance (ANOVA) followed by a post hoc test was used to identify significant differences between groups, with a significance level set at p<0.05. The mean hair growth ratio was calculated across all groups (NAR, MXD, NAR + MXD, and ethanol control) using Matlab software.

## RESULTS

### Effect of NAR on hair growth

**Figure 3:**
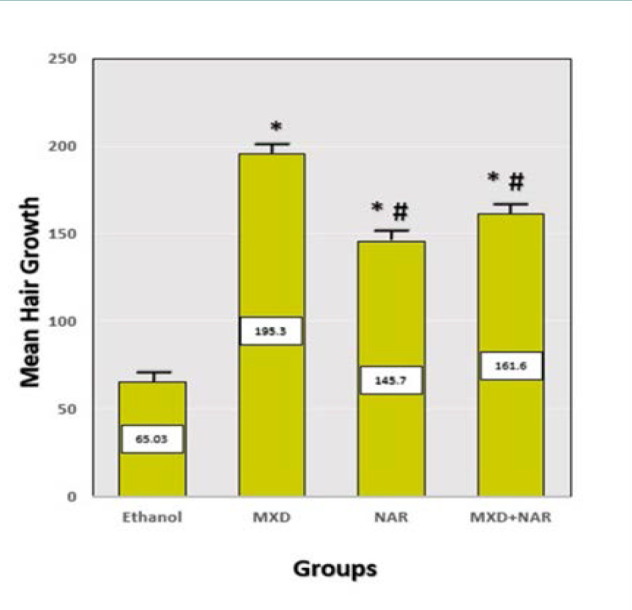
Effect of different treatments on hair growth Mean±SD, ***p-value<0.0001 vs. ethanol group, ## p-value<0.0001 vs. minoxidil group

Hair growth was enhanced in the NAR, MXD, and NAR + MXD treatment groups compared to the ethanol group (p<0.05) (Figure 3).

### Histological evaluation of hair follicles

**Figure 4:**
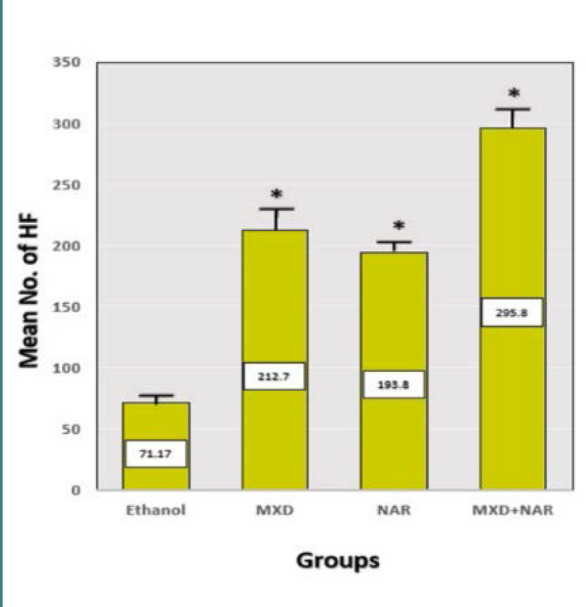
Effect of different treatments on the number of hair follicles Mean±SD, ***p-value<0.0001 vs. ethanol group, p-value<0.6 vs. minoxidil group

**Figure 5:**
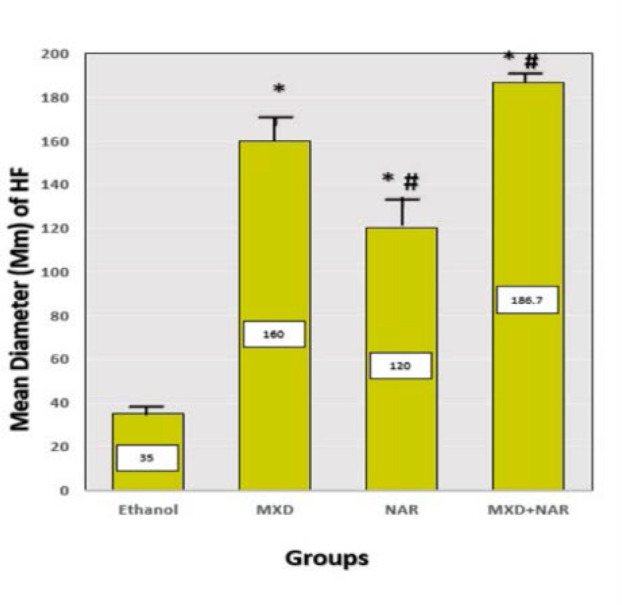
Effect of different treatments on the diameter of hair follicles Mean±SD, ***p-value<0.0001 vs. ethanol group, ## p-value<0.0008 vs. minoxidil group

When compared to the ethanol control group, the number of hair follicles increased considerably (p-value<0.05) in the NAR, MXD, and NAR +MXD groups (Figure 4). Histological analysis was used to calculate the average number of hair follicles in four test groups (each with six animals). The diameter of hair follicles also increased in all treatment groups, with a p-value of less than 0.05. (Figure 5). Hair follicle diameter (in micrometers) across four groups was measured by histological analysis at the conclusion of the research.

### NAR elevated the level of TAO-C in skin tissue

**Figure 6:**
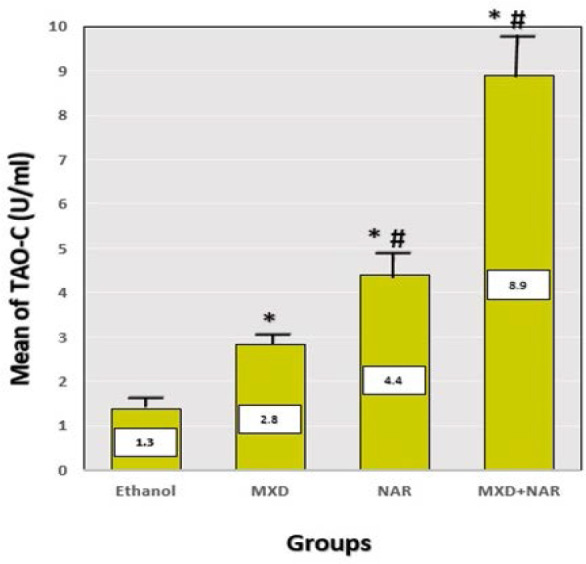
Effect of different treatments on total antioxidant capacity Mean±SD, ***p-value<0.0001 vs. ethanol group, ## p-value<0.0001 vs. minoxidil group

The results showed a significant elevation in the level of TAO-C within hair follicles among groups treated with NAR, MXD, and NAR+MXD (p-value<0.05) compared to the ethanol group (Figure 6). Hair follicle TAO-C levels were assessed using endpoint ELISA measurements and are presented as means in U/ml.

### Effect on NAR in combination with MDX on VEGF within hair follicles

**Figure 7:**
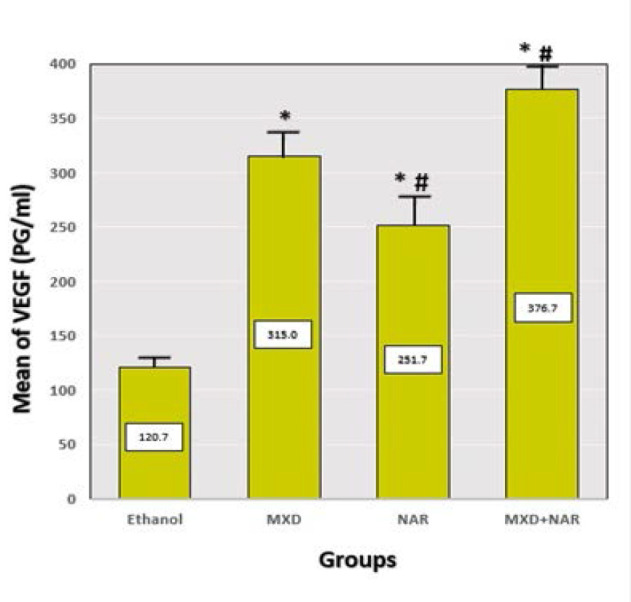
Effect of different treatments on VEGF levels within hair follicles Mean±SD, ***p-value<0.0001 vs. ethanol group, ## p-value<0.0006 vs. MXD group

The NAR, MXD, and NAR +MXD groups had a significant increase in VEGF levels within hair follicles (p-value<0.05) compared to the ethanol group (Figure 7). The mean VEGF levels within hair follicles were measured in picograms per milliliter (pg/ml) using ELISA.

### Effect of NAR and MDX on KGF within hair follicles

**Figure 8:**
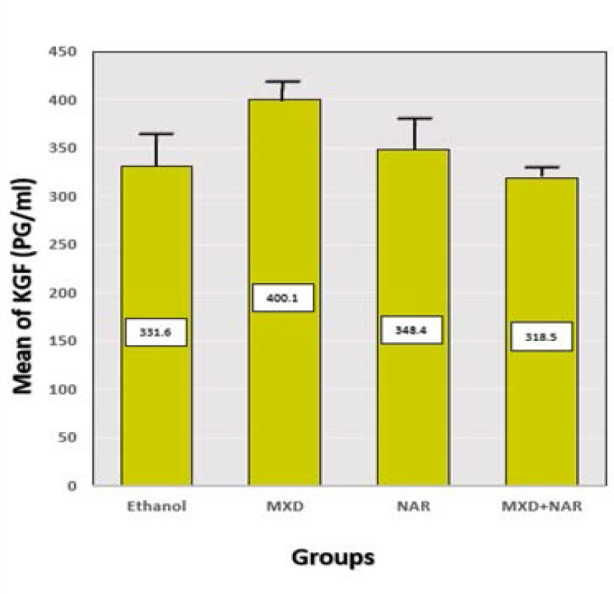
Effect of different treatments on KGF Mean±SD, p-value<0.9 vs. ethanol group; p-value<0.6 vs. MXD group

The results indicated a non-significant increase in the levels of the KGF marker within hair follicles among the groups treated with NAR, MXD, and NAR+MXD (p-value>0.05) when compared to the control group treated with ethanol (Figure 8). The average KGF levels in picograms per milliliter (pg/ml) were determined from hair follicles in the four groups.

## DISCUSSION

Hair loss, clinically known as alopecia, manifests as a reduction in hair density on the scalp, often more noticeable at the front of the head. In some cases, hair loss may affect only a small section of the body, while in others, it may affect the entire body [32]. Hair loss can be intermittent or persistent [25]. For some people, the loss of hair is a symptom of an underlying psychological disorder. Traditionally viewed as a cosmetic concern, especially in women, alopecia has significant psychological and social implications, underscoring the need for effective treatments with minimal side effects. NAR has pleiotropic mechanisms, which include antioxidant and anti-inflammatory actions. These mechanisms can potentially contribute to its effectiveness in managing hair loss or increasing hair growth [34].

### Effect of NAR on study parameters

#### Hair growth

Our study demonstrated that after 21 days of treatment, the NAR group significantly improved hair growth compared to the negative control group, although it was less effective than minoxidil. This aligns with one study that observed enhanced hair growth in human dermal papilla cells and keratinocytes treated with NAR [35]. Additionally, another study revealed that introducing various concentrations of NAR into keratinocyte cell culture mediums at 24, 48, and 72 hours appeared to promote hair development [34]. Their study showed a notable reduction in protein expression levels and cytokine gene expression in the NAR-treated keratinocyte cells compared to the control group. The 2,2-diphenyl-1-picrylhydrazyl assay also indicated a rapid decrease in absorbance, confirming the antioxidant activity of NAR. Furthermore, an *in vivo* assay highlighted the effectiveness of a gel formulation containing NAR in treating individuals with alopecia areata. When applied to the scalp for three months, this formulation showed promising results in managing this patchy hair loss condition [34]. Both *in vivo* and *in vitro* studies suggest that the beneficial effects of topical NAR on hair growth are dose-dependent.

#### Hair follicle number and diameter

Our research showed a significant rise in hair follicles in the group treated with NAR compared to the control groups (ethanol). However, this increase was not significant when compared to the minoxidil group after three weeks of treatment. Moreover, we observed a significant enlargement in the diameter of hair follicles in the NAR-treated group compared to the control group (ethanol). However, this was not significant when compared to the minoxidil group after three weeks of management. These findings support another study that discovered that applying NAR topically resulted in a significant increase in the number and diameter of hair follicles compared to the control groups [34]. Our study observed that the topical NAR application in mice leads to a significant proliferation and expansion of hair follicles, but the mechanism of action has not yet been clarified in detail. Additional experimental studies are needed to examine the stimulating effects of topical NAR on hair growth, both *in vitro* and *in vivo*.

#### TAO-C within hair follicles

This study revealed an important increase in TAO-C in the NAR group compared to the ethanol one. However, the increase in the level of TAO-C was higher in the minoxidil group. Our findings were consistent with Liang *et al*., who discovered that NAR significantly inhibited Bax and caspase-3 expression while simultaneously enhancing Bcl-2 expression. NAR increased the activity of key antioxidants such as SOD, GSH, and TAO-C when applied to serum from patients with pemphigus vulgaris. This indicates that NAR may block the Nucleotide-binding Oligomerization Domain 2 (NOD2)-mediated Nuclear Factor-kappa B (NF-κB) pathway, protecting keratinocytes from apoptosis and oxidative stress injury [36]. Also, Hermenean *et al*. [37] demonstrated the protective potential of NAR beyond dermatological applications. Their study found that oral pre-treatment with NAR in mice offered renal protection, effectively countering CCl4-induced oxidative damage and morphological injuries at the kidney level by conserving the endogenous antioxidant mechanism and scavenging free radicals.

#### VEGF and KGF in hair growth

VEGF levels were significantly higher compared to the ethanol group after 21 days of NAR therapy. However, the increase was less pronounced than in the minoxidil group. These findings align with another study, demonstrating that NAR stimulates VEGF secretion, supporting hair growth-associated angiogenesis. However, the stimulatory effects of topical NAR were demonstrated only *in vitro* [35]. In addition, NAR increased the viability of hypoxic human umbilical vein endothelial cells (HUVECs), as well as the abilities of tube formation and migration, and further inhibited the expression of miR-223-3p during *in vitro* experiments. Thus, NAR improved the angiogenesis of HUVECs by regulating the miR-223-3p/IGF1R axis, suggesting that it has the potential to promote angiogenesis in mice with myocardial infarction [38]. This stimulatory action of NAR on VEGF indicates its effectiveness as a hair growth promotor. Additional experimental studies are needed to examine the stimulating effects of topical NAR *in vitro* and *in vivo* models of hair growing. Furthermore, the current study showed an insignificant effect on the tissue level of KGF after 21 days of NAR treatment compared to the minoxidil and control (ethanol) group. There was no evidence that NAR had an effect on KGF level on hair growth. However, Liang *et al*. suggested naringenin protected HaCaT cells from PV serum-induced apoptosis. Also, NAR downregulated the expression of keratinocyte adhesion molecules and inhibited the disruption of cell–cell contacts in HaCaT cells [36].

### The combined effect of naringenin and minoxidil on hair growth parameters

Our study demonstrated that the topical application of a combination of NAR and minoxidil significantly improved hair growth, increased the diameter of hair follicles, and elevated the tissue levels of TAO-C and VEGF activity. However, it had no effect on tissue levels of KGF following three weeks of treatment compared to negative control and minoxidil-only groups. The combination of NAR and MXD had no significant effects on the number of hair follicles compared to minoxidil-alone. On the other hand, the combined NAR and MXD exhibited a significant increase in the number of hair follicles compared to the negative control. To our knowledge, this is the first study to investigate the combined effects of NAR and MXD on hair growth. Our findings suggest that the combination of these two agents may exert stimulating effects on hair follicles, contributing to their hair growth-promoting action.

## CONCLUSION

Overall, the data from our study collectively indicate that naringenin, with its antioxidant and anti-inflammatory properties, plays a significant role in mitigating hair loss in adult male mice. These findings underscore the potential of NAR as a therapeutic agent in treating hair loss, particularly due to its ability to slow down the process.
